# Round the Hospitals

**Published:** 1916-03-11

**Authors:** 


### Round the Hospitals.

We are glad to know that The Hospital reaches some, at any rate, of the large and increasing number of sisters who have been called up for military service. Their collective experiences will no doubt cover the whole field of nursing, a fact the mention of which manifests the never-ending variety and interest of each day's work. Those of us whose duty confines them to a less exciting but no less arduous field in association with the hospital care of the wounded in the homeland, and the war problems which incidentally present themselves for solution, would welcome news from every sister whose work may lie in more exciting fields nearer the war centres. We look forward with hope to the arrival of briefly written items of news of the kind, for they should help to spread contentment amongst the less fortunate but continuously occupied home workers.

The following most interesting and helpful information has been sent us from the Norfolk and Norwich Hospital. It is a tribute to the industry and working powers of the sister-housekeeper there, and deals with the work which has been done in spare minutes and by odd maids in a busy kitchen. In 1915, 2,000 lb. of marmalade, 4,000 lb. of jam, and enough pickles and chutney to supply the needs of the patients and staff for a whole year were made there under the direction of the sister-housekeeper. Exclusive of the cost of gas, the marmalade cost a fraction over 2d. a lb., whilst the jam cost less than 2d. a lb., some of the fruit being given. The bottled fruits were sterilised by steam, the only extra being about one tablespoonful of sugar to 4 lb. of fruit, the prime cost being only the wholesale price of the fruit, which was purchased in the early hours of the morning at the weekly provision market. Another practical and useful economy has been the organisation and employment of a considerable number of voluntary workers in the laundry. By their aid it has been possible to work the laundry efficiently without extra hands, despite the large addition to the work incidental to the treatment of a number of military patients.

---

We welcome the co-operation of Miss Darbyshire, the matron of St. Mary's Hospital, who has kindly arranged with one of her sisters to act as correspondent for this column. Miss Darbyshire takes a deep interest in the important question of what will be the position of trained nurses at the end of the war. This subject was exhaustively dealt with in the "Nursing Outlook" in the *Nursing Mirror* of January 1, wherein the necessity for the immediate unity of nurse-training schools was insisted upon. It was still more convincingly dealt with in Sir Charles Russell's speech published in The Hospital last week, and in the "Nursing Outlook" in the *Nursing Mirror* of the 4th instant. Miss Darbyshire's views were communicated to us only a fortnight ago, and in that short interval events have happened which we feel confident will ensure the uniting of the overwhelming majority of the ablest minds and the most knowledgeable leaders of the nursing profession in these islands.

---

Information and suggestion seem to be widely desired on the administrative side of a ward sister's work. It is held that a few practical notes dealing with this aspect of the inner daily life of a great hospital would prove beneficial. We wish ward sisters who read this column habitually would give us their experience as to the attitude of ward sisters on this side of a sister's work. For instance, how many sisters in fact take an intelligent interest in the expenditure which is indirectly and sometimes directly governed by the efficiency of the ward sister? On the question of expenditure, does the training of the sister, of the charge nurse, and of her probationers cover insistence upon the duty of continuous watchfulness as to the adequate regulation of the consumption of gas in stoves and of electricity in lighting? Take again the waste, and also the loss through damage, in linen and all articles passing between the ward and the laundry; in the receipt, distribution, and custody of stores; in the issue and careful use of surgical dressings. There is the art of serving the meals. It is a golden rule to give a patient rather less than more in the first helping, because this plan encourages appetite and gives cheerfulness to the patient if he gets a second helping when able to consume it. In all these ways and many others a ward sister who has studied the administrative side of her work and puts in practice her knowledge in regard to it can save sometimes in a month, always in a twelvemonth, the whole of her salary. We gave some suggestive information on this head in The Hospital of October 16, 1915, page 57. Matrons might help their sisters if they had a copy of the new edition (now in the press) of the "Uniform System of Accounts for Hospitals and Institutions," published by The Scientific Press, 28 and 29 Southampton Street, Strand, W.C.

---

Referring to the splendid war record of the Royal Infirmary, Liverpool, given in The Hospital of the 26th ultimo, we regret that the total number of beds, 330, in the hospital was wrongly given as the number devoted to wounded soldiers, which number is seventy-two.

---

Certainly all workers for the sick throughout the Empire may be cheered by the reflection that their personal service, fulfilled in the right spirit, is far above all material considerations and the glamour of the limelight. The faith which animates individual workers makes them indifferent to worldly considerations, for the spiritual should always predominate in work for the sick and injured. This is not only a case of workers in children's hospitals but of workers everywhere in the humblest positions in the smallest institutions, apart altogether from military hospitals and military excitement.

---

The matron, whose thoughts on the bestowal of honours to workers in the nursing field we published two weeks ago, makes a suggestion in regard to the rank and file of the nursing profession, which she thinks may possibly be already in the minds of the authorities. This suggestion is that every individual worker should receive a medal at the end of the war, whether she may have attended the wounded at home or abroad. "In this way the work of all would be equally recognised, and those whose privilege it has been to serve abroad would not have the additional advantage of gaining honours, while those who have worked at home were passed over. In this connection I should like to quote a passage from a book I have just been reading ('Recollections and Reflections,' by Bishop Welldon): 'The multiplication of prizes has tended to lower the dignity of English character. It naturally engenders a passion for honours. . . . Nobody, it seems, can perform a meritorious action but he wishes to see it blazoned in the newspapers. The golden rule of "not letting" the "left hand know what" the "right hand doeth" has largely fallen into desuetude, if not into contempt. But the product of manhood and womanhood, which is the highest educational product [and might we not say nursing product?], is independent of external recognition; it is ready and proud to render services without seeking praise for them; it finds a sufficient reward in the sense of having rendered them; it knows that the supreme virtue lies in doing good, not only without earthly recompense, but to the unthankful and the evil.'" We shall be very glad to have the views of our readers on the points here raised.

After the war there will be a lamentable deficiency in the number of trained nurses available, a fact which we commend to the consideration of Liverpool men, many of whom are reputed to be making huge fortunes, that they may generously turn their attention to the great Royal Infirmary in their midst by inspecting the present inadequate accommodation there for the nursing staff, and providing the funds immediately to erect a nursing home of adequate proportions, and to make it so well equipped and so efficient as to constitute a unit for the helpful consideration of other great institutions throughout the country. Liverpool men should remember in this connection that the home at the Liverpool Royal Infirmary is one of the oldest in England; that it has cost the present generation nothing; that the institution deservedly has the highest reputation for efficiency throughout the North of England; and that the present generation has done nothing really to provide adequately for the nursing staff or to make it worthy of the wealth and position of the second city in the United Kingdom. In this connection we may congratulate the Liverpool authorities upon the appointment of Miss L. Atkinson, the assistant matron at the Royal Infirmary and former senior home sister, to the important post of matron to the Northampton General Hospital. We also congratulate them on that of Miss Owston, their housekeeping sister, to be matron of the Baldonan Institution for Children, near Dundee.

---

It is our invariable practice to pay for all items of news which we may publish. The minimum payment is 5s. Paragraphs of about twenty lines in length—that is to say, of 150 words or less—are preferred. In this section during the war we propose to give 10s. to the correspondent who may send us in any week the best incident connected with hospital life and work. In this way we hope to afford to all practical aid and encouragement; while those who reciprocate will be able to render us invaluable service. Contributions should be addressed to The Editor, The Hospital, 28 and 29 Southampton Street, Strand, London, W.C., and, to be in time for the current week's issue, should reach him by the first post on Mondays.

